# 621. Evaluation of a Multiplex Endpoint PCR for the Detection of 21 Fungi Commonly Causing Fungal Pneumonia Using Barcoded Magnetic Bead Technology

**DOI:** 10.1093/ofid/ofad500.687

**Published:** 2023-11-27

**Authors:** Katharine Uhteg, Kristen Haag, Michael Aye, Martín Cardona, Warda Memon, Marissa Totten, Sean Zhang

**Affiliations:** Johns Hopkins Hospital, Baltimore, Maryland; Applied BioCode, Santa Fe Springs, California; Applied BioCode, Santa Fe Springs, California; Applied BioCode, Santa Fe Springs, California; Johns Hopkins Hospital, Baltimore, Maryland; Johns Hopkins University, Baltimore, Maryland; Johns Hopkins University, Baltimore, Maryland

## Abstract

**Background:**

Fungal pneumonia caused by *Aspergillus*, Mucorales, and other fungi are potentially life-threatening and often are difficult to diagnose due to similar clinical presentations and poor sensitivity by culture. The BioCode MDx-3000 from Applied BioCode uses Barcoded Magnetic Bead (BMB) technology to digitally detect 21 fungi in a multiplex PCR. We evaluated the BioCode assay using clinically simulated samples and retrospective frozen bronchoalveolar lavage samples.

**Methods:**

For limit of detection (LOD), 10-20 replicates of each organism were run at 1E4-10 spores/mL. Reproducibility was performed by running three replicates of each target (1E3 spores/mL) within one run, three separate times. Specificity was determined by testing for 37 fungal analytes not targeted by the panel. Matrix specific inhibition was also tested by spiking each organism in the following matrices: whole blood, plasma, serum and BAL. 90 retrospective BAL samples from patients with fungal culture results were run to assess clinical performance.

**Results:**

LOD of 21 fungal organisms ranging from 19.83 – 271.76 spores/ml. Detection rate was 100% (36/36) in all samples mixed with two fungi; 95% (20/21) in all samples mixed with three fungi. No detection or cross-reactivity was observed in samples containing any of the 37 non-targeted fungi. All replicates were detected in Inter- and intra- reproducibility experiments. No difference or inhibition was observed in detection of targeted fungi spiked in different matrices. Testing retrospective BAL samples showed an overall positive concordance of 61.4% and negative concordance of 98.1%. In 23 BALs positive by both calcofluor white stain (hypha elements) and culture, the BioCode assay correctly detected fungal targets in 78% (18/23) of the samples. Additionally, the assay produced positive detection in 25% of BALs that were culture negative.

Limit of Detection and Concordance

**Figure:**
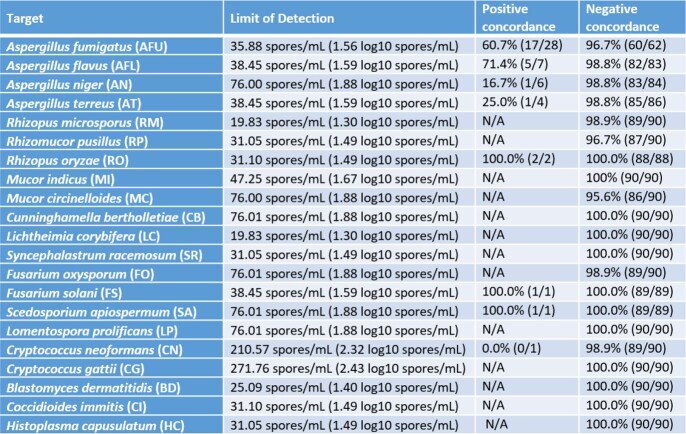

Limit of detection for the 21 fungal targets. Concordance for each target in retrospective BALs compared to culture. N/A indicates not tested.

**Conclusion:**

The BioCode assay showed an overall good analytical sensitivity and specificity. Analysis of retrospective BAL samples demonstrated the assay showing good concordance with the culture results. Further studies are warranted to determine the assay’s clinical sensitivity and specificity in aid in diagnosis of fungal pneumonia.

**Disclosures:**

**Kristen Haag, BS**, Applied BioCode, Inc.: I am an employee of Applied BioCode|Applied BioCode, Inc.: Stocks/Bonds **Michael Aye, PhD**, Applied BioCode: Stocks/Bonds **Martín Cardona, BS**, Applied BioCode, Inc.: I am an employee of Applied BioCode. **Sean Zhang, PhD**, Applied BioCode: Grant/Research Support|IMMY Diagnostics: Grant/Research Support|KARIUS: Advisor/Consultant|Pearl Diagnostics: Grant/Research Support|Scanogen: Grant/Research Support|T2 Biosystems: Advisor/Consultant|Vela Diagnostics: Grant/Research Support

